# Novel insights into irritability: the relationship between subjective experience, age and mood

**DOI:** 10.1192/bjo.2021.1033

**Published:** 2021-10-28

**Authors:** Erica Bell, Gin S. Malhi, Zola Mannie, Philip Boyce, Richard Bryant, Maree Inder, Richard J. Porter

**Affiliations:** Academic Department of Psychiatry, Kolling Institute, Northern Clinical School, Faculty of Medicine and Health, University of Sydney, Australia; and CADE Clinic, Royal North Shore Hospital, Northern Sydney Local Health District, Australia; Academic Department of Psychiatry, Kolling Institute, Northern Clinical School, Faculty of Medicine and Health, University of Sydney, Australia; and CADE Clinic, Royal North Shore Hospital, Northern Sydney Local Health District, Australia; Academic Department of Psychiatry, Kolling Institute, Northern Clinical School, Faculty of Medicine and Health, University of Sydney, Australia; CADE Clinic, Royal North Shore Hospital, Northern Sydney Local Health District, Australia; and NSW Health, Royal North Shore Hospital, Northern Sydney Local Health District, Australia; Speciality of Psychiatry, Sydney Medical School, Faculty of Medicine and Health, University of Sydney, Australia; School of Psychology, University of New South Wales, Australia; Department of Psychological Medicine, University of Otago, New Zealand; Department of Psychological Medicine, University of Otago, New Zealand

**Keywords:** Bipolar affective disorders, phenomenology, depressive disorders, nosology, psychosocial interventions

## Abstract

**Background:**

The relationship between irritability as a subjective experience and the behavioural indicators typically used to measure the construct are not known. Its links to mood, and contextual relationships, vary with age and are yet to be thoroughly examined.

**Aims:**

First, to interrogate the relationship between the subjective experience of irritability and mood, and that with its behavioural indicators. Second, to determine how these relationships vary with age and over time.

**Method:**

This study examined data from a previous clinical trial of adolescents and young adults (*N* = 82) with bipolar disorder, who received a psychological intervention over 18 months. Participants completed a battery of questionnaires, which included assessments of irritability. Analyses of covariance were conducted to examine the interaction between mood symptoms, subjective measures of irritability, behavioural measures of irritability and age over time.

**Results:**

Subjective irritability scores differed significantly over time when controlling for manic, but not depressive, symptom scores. Further, subjective irritability significantly differed when controlling for behavioural measures of irritability (temper outbursts and argumentativeness). There were significant interactions between scores of depressive symptoms, temper outbursts and subjective irritability with age, wherein younger participants showed no correlation between depressive symptoms and temper outbursts. In addition, younger participants showed lower correlations between subjective irritability and both depressive and temper outburst scores, than older participants.

**Conclusions:**

Subjective irritability is linked to mood morbidity and behavioural outbursts, and these relationships are contingent on age. Our novel findings suggest that subjective irritability should be assessed in greater detail in patients with mood disorders.

Irritability features across psychiatric disorders, throughout the lifespan, and contributes to significant functional impairment.^1^ It plays a particularly central role in mood disorders characterising both mania and depression, and at the same time, worsening prognosis and increasing the risk of suicide.^2–4^ Despite its seeming ubiquity and impact, irritability remains poorly understood and lacks an operational definition that would allow both valid and reliable measurement.^1,5,6^ This is partly because there is a lack of consensus as regards the essential components that should be included in an optimal measure of irritability and its causal determinants are poorly understood, as are the subjective experience of being irritable and its behavioural consequences.^1,5^

Irritability is often assessed through behavioural outcomes or observer-rated scales of irritability that assess temper outbursts, aggression and argumentativeness, especially in children and adolescents.^7,8^ But it remains unclear to what extent these external ‘proxies’ reflect the subjective experience of irritability. If this knowledge could be gained, it would inform the future development of much-needed rating scales that may then be able to meaningfully quantify irritability and allow comparisons across populations and different time points.^1^ Furthermore, although clinically, irritability appears in both ‘poles’ of mood (i.e. depression and mania), it only signifies depression in children and adolescents (aged <18 years), whereas in adults it defines mania.^9^ Thus, examining irritability in bipolar disorder, which spans both poles of the mood spectrum, and in particular across adolescents and young adults within this period of classificatory transmogrification, will likely yield useful insights into the relationships between irritability and other mood symptoms and its role in clinical psychopathology. Thus, at present, there are two pressing questions: first, where to look, and second, what to use to measure irritability?

Fundamentally, irritability is poorly understood. It is not known, for example, whether the phenomenon occurs as a direct consequence of an underlying mood disorder, or whether it manifests as an epiphenomenon alongside mood symptoms.^10–12^ Given that irritability is a core feature of mood disorders, this is a logical population to examine, and understanding the relationship between subjective irritability and the emotional context within which it manifests should yield critical insights into the mechanisms underlying both mood disorders and irritability. At the same time any such insights may help inform the future development of a comprehensive and validated measure of subjective irritability, which in turn, may furnish us with a more granular appreciation of its relationship with potential behavioural indicators.

## Aims

Therefore, in this exploratory study, we aimed to examine the relationship between subjective ratings of irritability and scores of mood symptom severity (depression and mania), having hypothesised that these are likely to vary depending on the polarity of mood.

In addition, we also interrogated the relationships between subjective measures of irritability, and its behavioural proxies (such as temper outbursts and argumentativeness), having theorised on the basis of clinical experience that these may vary according to age.

## Method

### Procedure

In this study, we compared measures of irritability using data from a study of adolescent and adult patients diagnosed with a mood disorders enrolled in a psychological therapy trial.^13^ This trial data was chosen specifically, as it includes questions that interrogate irritability in detail, including its subjective and behavioural indicators. This data was also readily accessible and included adolescents for whom the relationship between irritability and mood symptoms was of particular interest. Detailed methodology is provided in the publication of this randomised controlled trial of interpersonal and social rhythm therapy (IPSRT) versus a ‘control’ psychological intervention of specialist supportive care (SSC) over 18 months.^13^ The authors assert that all procedures contributing to this work comply with the ethical standards of the relevant national and institutional committees on human experimentation and with the Helsinki Declaration of 1975, as revised in 2008. Ethical consent for the original study and any subsequent analysis was obtained from the Canterbury Ethics Committee, and the trial was registered with the Australian and New Zealand Clinical Trials Registry (identifier ACTRN12605000722695). Here, we summarise the key aspects pertinent to the current analysis.

The measures included self-report and clinician-rated measures that examine both the subjective experience and possible behavioural consequences of irritability. Participants completed each questionnaire at ten time points every 9 weeks, over the 78-week study. Therefore, measurements were taken at weeks 0 (baseline), 8, 17, 26, 34, 43, 52, 61, 69 and 78. Comparisons were conducted over the course of the study, to assess their potential validity as measures of irritability at different ages and over time.

This data was selected because the population sampled was homogeneous with regard to diagnosis (bipolar disorder) and participants had been assessed longitudinally at frequent intervals as their mood symptoms improved. In addition, the sample included participants aged 15–36 years, and this enabled further examination of the links between behavioural and subjective measures across an important developmental period within adolescence that extends into early adulthood. Thus, this data is particularly useful as it provides a unique opportunity to observe the relationship between assessments of mood and irritability over time.

### Participants

The study recruited 100 adolescents and young adults aged 15–36 years with bipolar disorder type 1, bipolar disorder type 2 and bipolar disorder not otherwise specified, with an adequate understanding of English, to participate in psychotherapy. Written informed consent was obtained from all patients. Participants were recruited from a range of services, including mental health services and general practitioners. Exclusion criteria were schizophrenia or schizoaffective disorder and severe alcohol or drug dependence. Of the 100 patients recruited, 84 completed the study. Across both treatment groups, the reasons for failure to complete were: moving from the study location (*n* = 6), failure to engage (*n* = 1), withdrawal (*n* = 3), requiring alternative treatment (*n* = 2), death by suicide (*n* = 1) and lost to contact (*n* = 3).^13^ Of this group, 82 had complete data-sets and were included in the final analyses.

### Clinical assessment

After obtaining consent and before randomisation, the treating psychiatrist completed an Axis I diagnostic assessment, using the Structured Clinical Interview for DSM-IV.^14^ Mood symptom severity was rated with the Montgomery–Asberg Depression Rating Scale (MADRS)^15^ and the Young Mania Rating Scale (YMRS).^16^

Participants also completed a modified version of the self-report Symptom Checklist-90 (SCL90), a 90-item comprehensive psychological assessment of a broad range of psychopathology.^17^ This assessment asks ‘During the past week, how much were you bothered by … ’ for each item listed, which can be scored from 0 (not at all) to 4 (extremely). The hostility subscale^18^ includes an irritability item (item 11 – ‘Feeling easily annoyed or irritated’), as well as behavioural proxies. These ‘proxy items’ include items 24, 63, 67, 74 and 81. In addition, the modified item of 76a was included in the proxy items as it shared phenomenological overlap with other hostility items (see Table 1).

**Table 1:** Hostility subscale items within the Symptom Checklist-90

Item number	Item
11	Feeling easily annoyed or irritated
24	Temper outbursts that you could not control
63	Having urges to beat, injure or harm someone
67	Having urges to break or smash things
74	Getting into frequent arguments
76a	Argumentative
81	Shouting or throwing things

### Statistical analysis

All analyses were performed with IBM SPSS Statistics^19^ (version 26 for Windows). First, we examined differences between treatment groups at baseline and follow-up (confirmation of previous results reported in this cohort), using *t*-tests. This determined whether to separate participants according to treatment received during the study, or to examine the patients as one group. Second, we examined the univariate correlations between mood and irritability items at baseline. This indicated whether a significant relationship existed between irritability and mood scores at baseline before continuing with the analysis over the course of the study.

Finally, to interrogate how age modifies the relationship between behavioural and subjective measures of irritability, the interaction between age and the comparison variable of interest was examined. As we aimed to examine the impact of mood symptoms, behavioural indicators of irritability, age and the interaction between these factors and time on subjective irritability scores, analyses of covariance (ANCOVAs) were used to interrogate these relationships. In line with previous results reported in this cohort, parametric statistical tests were utilised, as scores for each variable were found to be normally distributed over the duration of the study.

Thus, we used ANCOVA to examine the relationships between SCL90 item 11 (as the dependent variable) and either total mood scores (YMRS or MADRS) or other individual SCL90 item scores as the covariate (to examine the relationship between mood and behavioural proxies, respectively), with age as an additional covariate. Of note, the time points for assessment were included as a fixed variable (time) and participant identification number as a random factor. Finally, the model was used to examine each main effect, as well as two interactions, one between the comparison variable and age and the second between the comparison variable and time. All assumptions to perform ANCOVA were satisfied.

## Results

There were no significant differences in scores of the SCL90 item11, YMRS or MADRS between treatment groups (IPSRT and SSC) at baseline or at the conclusion of the study.^13,20^ This is in keeping with the findings of earlier analyses that have been published as part of the results of the clinical trial. However, total scores on the SCL90 item 11 and MADRS across all participants did significantly differ between baseline and week 78 (i.e. after long term IPSRT or SSC) (see Table 2). Therefore, in our subsequent analyses, participants were not separated according to the psychological treatment that they had received during the study.

**Table 2:** Descriptive statistics of irritability, mania and depression symptom scores

	Baseline	*n*	Week 78	*n*	*t*
YMRS (s.d.)	2.21 (3.56)	82	1.33 (2.63)	82	1.787
MADRS (s.d.)	14.67 (10.87)	82	7.65 (9.35)	82	4.489**
SCL90 item 11 (s.d.)	1.75 (1.33)	80	0.95 (1.19)	80	4.032**

Variation in *n* is a result of incomplete measures of SCL90 at week 78. YMRS, Young Mania Rating Scale; MADRS, Montgomery–Asberg Depression Rating Scale; SCL90, Symptom Checklist-90.

** *P* < 0.01 (two-tailed).

At baseline, Pearson's correlations analyses showed a significant correlation between scores on the total YMRS, total MADRS and SCL90 item 11 (irritability) (see Table 3).

**Table 3:** Pearson correlation matrix between irritability, mania and depression symptom scores at baseline

	1. SCL90 irritability	2. YMRS total	3. MADRS total
1. SCL90 irritability	1		
2. YMRS total	0.51**	1	
3. MADRS total	0.355**	0.020	1

SCL90, Symptom Checklist-90; YMRS, Young Mania Rating Scale; MADRS, Montgomery–Asberg Depression Rating Scale.

** *P* < 0.01 (two-tailed).

### Comparisons between irritability and mood morbidity

An ANCOVA was performed to determine if there was a statistically significant change in scores on the SCL90 item 11 (irritability) across the 78 weeks (time), controlling for age and scores on the YMRS. There was a significant main effect of time on irritability scores (F(1, 672) = 2.855, *P* = 0.003). There was no main effect of YMRS score on irritability scores (F(1, 672) = 0.974, *P* = 0.324). There was no significant interaction between age and YMRS score on irritability scores (F(1, 672) = 0.847, *P* = 0.358) and there was no interaction between time and YMRS scores on irritability (F(9, 672) = 0.419, *P* = 0.890).

Similarly, an ANCOVA was also conducted to determine if there was a statistically significant difference in scores of SCL90 item 11 (irritability) over the 78 weeks (time), controlling for age and scores on the MADRS. There was no significant main effect of MADRS scores on irritability scores (F(1, 672) = 0.001, *P* = 0.982). There was no significant main effect of time on irritability scores (F(9, 672) = 1.515, *P* = 0.139). There was a significant interaction between age and MADRS scores on irritability scores (F(1, 672) = 5.043, *P* = 0.025). There was no significant interaction between time and MADRS scores on irritability scores when controlling for age (F(9, 672) = 0.823, *P* = 0.595).

### Items within the SCL90

To examine the relationship between irritability scores and scores on behavioural proxy items of irritability, a series of ANCOVAs were conducted to determine if there was a difference in irritability scores of SCL90 item 11 (irritability) over the 78 weeks (time), controlling for age and scores of the behavioural proxy items in the SCL90 (see Table 4). In all analyses, there was a significant main effect of time on irritability scores (all *P* < 0.001) when controlling for the other SCL90 hostility items. In all analyses, there were no significant interactions between SCL90 hostility items and time on irritability scores when controlling for age. The relationships between the items are detailed below.

**Table 4:** Interactions between mood and irritability variables with age and time

Comparison variable	Interaction with age	Interaction with time
YMRS	F(1, 672) = 0.847	F(9, 672) = 0.477
MADRS	F(1, 672) = 5.043*	F(9, 672) = 0.823
SCL90 items		
24	F(1, 673) = 6.594**	F(9, 673) = 0.847
63	F(1, 674) = 0.378	F(9, 674) = 1.418
67	F(1, 674) = 0.015	F(9, 674) = 0.452
74	F(1, 674) = 0.628	F(9, 674) = 0.674
76a	F(1, 674) = 0.159	F(9, 674) = 0.611
81	F(1, 674) = 0.271	F(9, 674) = 1.635

YMRS, Young Mania Rating Scale; MADRS, Montgomery–Asberg Depression Rating Scale; SCL90, Symptom Checklist-90.

* *P* < 0.05.

** *P* < 0.01.

There was a main effect of SCL90 item 24 (temper outbursts) (F(1, 673) = 26.293, *P* < 0.001), item 67 (having the urge to break or smash things) (F(1, 674) = 5.146, *P* = 0.024), item 74 (getting into frequent arguments) (F(1, 674) = 9.282, *P* = 0.002) and item 76a (argumentative) (F(1, 674) = 15.509, *P* < 0.001) scores, on SCL90 item 11 (irritability) scores. There was no main effect of item 63 (F(1, 674) = 0.171, *P* = 0.679) or item 81 (F(1, 674) = 3.118, *P* = 0.078) scores on irritability scores.

There was a significant interaction between item 24 scores and the age of participants on scores of subjective irritability (F(1, 673) = 6.594, *P* = 0.010). There were no significant age interactions between age and item 63 (F(1, 674) = 0.378, *P* = 0.539), item 67 (F(1, 674) = 0.015, *P* = 0.904), item 74 (F(1, 674) = 0.628, *P* = 0.429), item 76a (F(1, 674) = 0.159, *P* = 0.690) or item 81 (F(1, 674) = 0.271, *P* = 0.603) scores, on irritability scores.

### Relationships with age

As scores on both the MADRS and SCL90 item 24 showed significant age interactions, Pearson's correlations analyses (with Bonferroni correction) were conducted with participants split into four groups: those aged 15–20 years (*n* = 16), those aged 21–25 years (*n* = 20), those aged 26–30 years (*n* = 14) and those aged 31–36 years (*n* = 32). These correlations showed that scores of temper outbursts (SCL90 item 24) were not correlated with MADRS scores in those aged 15–20 years, but that there was a correlation in all other age groups and in particular, was a strong correlation in those aged 21–25 years (see Table 5). In addition, subjective irritability (SCL90 item 11) scores were more closely correlated with SCL90 item 24 scores (temper outbursts) in those aged ≥21 years, relative to those aged 15–20 years.

**Table 5:** Pearson's correlation matrix between irritability, MADRS and SCL90 item 24 scores in participants divided according to age

First quartile (15–20 years) (*n* = 16)			
	1. SCL90 item 11 (irritability)	2. MADRS total	3. SCL90 item 24
1. SCL90 item 11 (irritability)	1		
2. MADRS total	0.325**	1	
3. SCL90 item 24	0.405**	0.064	1
Second quartile (21–25 years) (*n* = 20)			
1. SCL90 item 11 (irritability)	1		
2. MADRS total	0.591**	1	
3. SCL90 item 24	0.669**	0.459**	1
Third quartile (26–30 years) (*n* = 14)			
1. SCL90 item 11 (irritability)	1		
2. MADRS total	0.377**	1	
3. SCL90 item 24	0.562**	0.296**	1
Fourth quartile (31–36 years) (*n* = 32)			
1. SCL90 item 11 (irritability)	1		
2. MADRS total	0.524**	1	
3. SCL90 item 24	0.589**	0.262**	1

Montgomery–Asberg Depression Rating Scale; SCL90, Symptom Checklist-90.

** Correlation is significant at *P* < 0.01 (two-tailed).

## Discussion

This exploratory study aimed to examine the relationship between clinical measures used to assess irritability and mood in a sample of adolescent and adult patients with mood disorders. In line with our hypotheses, subjective irritability scores appeared to be significantly different when controlling for scores across other domains of irritability, such as proxy measures of behavioural changes. However, contrary to our hypotheses, measures of mood morbidity did not correlate with measures of irritability over the duration of the study. Furthermore, the relationships between these measures of irritability, behavioural proxies and mood varied according to age.

When examining the relationship between subjective irritability scores (SCL90 item 11) and those of overall mood morbidity, irritability scores significantly differed over time when controlling for YMRS, but not MADRS scores. Within the SCL90, irritability scores (item 11) did significantly differ when controlling for items 24 (temper outbursts), 67 (urge to break or smash things), 74 (getting into frequent arguments) and 76a (argumentative), over time. When examining interactions between measures of mood, irritability and age, there were significant interactions between MADRS and SCL90 item 24 with age, to show a significant difference in irritability scores (item 11) over the 78 weeks.

When examining further the relationship between irritability, MADRS and SCL90 item 24 scores, and age, the participants were split into four age groupings. Participants aged 15–20 years showed no correlation between MADRS and item 24 scores (temper outbursts); however, this correlation was significant in all other age groupings (21–36 years). In addition, participants aged 15–20 years showed lower correlations between subjective irritability and both MADRS and item 24 scores, than older participants. Participants aged 21–25 years showed the strongest correlations between subjective irritability, MADRS score and item 24 score.

### Mood morbidity

One reason why YMRS scores were not a significant covariate in scores of subjective irritability over time, may be because these did not significantly change from baseline to week 78. In addition, as baseline scores for the YMRS were relatively low to begin with, this did not leave much scope for any further significant decrease over the course of the study, and this made detecting any potential relationship between these scores and those of subjective irritability difficult. Future studies should compare subjective irritability and objective irritability in patients with higher levels of manic symptoms to examine whether the findings are applicable to those with more acute symptoms of mania.

This finding is mirrored in MADRS scores, wherein depressive symptoms are also correlated with irritability at baseline, but again, does not significantly correlate with these irritability scores over time. Interestingly, this relationship differed by age, where participants aged 21–36 years, and in particular those aged 21–25 years, showed a stronger relationship between MADRS scores and irritability scores. This is an intriguing finding, as irritability is typically assumed to be a core feature of depression in children and adolescents, and is categorised as such in major classification systems.^9^ This finding may indicate that irritability plays a different role in depression depending on age, wherein it is more central to typical depressive symptoms (as measured by the MADRS) in older individuals. Additionally, it may suggest that subjective irritability plays a different role in younger people, and its manifestation in the context of depressive symptoms may be less apparent, or different from that observed in older individuals.

We believe the differential relationship between irritability and depressive symptoms according to age is important because it potentially holds key insights as regards the experience of irritability in mood disorders.

Currently, it is unknown whether irritability experienced within the context of mood disorders occurs as a direct symptom of the illness, or as an epiphenomenon that coincides with the illness but is not a direct consequence *per se*.^11,12,21^ It may be that the subjective experience of irritability varies in accordance with the mood state within which it occurs, i.e. mania or depression. This suggests an interaction between irritability and emotion, such that the subjective appraisal of irritability is affected by the emotion of a mood state caused by an illness such as bipolar or unipolar depression. Thus, the findings of the present study reveal novel, differential relationships between irritability, mood scores and age, which should be interrogated further in future studies.

### SCL90

**Fig. 1:**
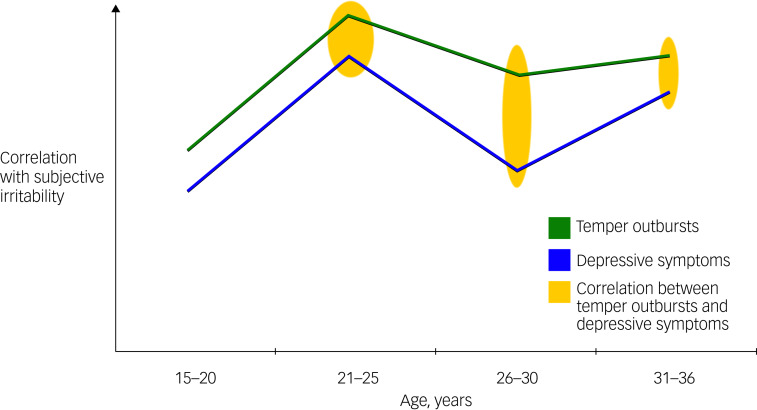
Schematic illustrating differential relationship between symptoms according to age. This schematic illustrates how temper outbursts (SCL 90 item 24, green) and depressive symptoms (MADRS, blue) correlate more strongly with subjective irritability (SCL90 item 11) in those aged 21–36 years. Furthermore, this schematic also shows the correlation between temper outbursts and depressive symptoms (yellow) in those aged 21–36 years, which is not present in those aged 15–20 years. This correlation was strongest in those aged 21–25 years, as indicated by the thickness of the yellow ovals. MADRS, Montgomery–Asberg Depression Rating Scale; SCL90, Symptom Checklist-90.

Comparisons of the subjective scores of irritability, with the behavioural proxy items listed within the hostility subscale of the SCL90, identified some key relationships. First, most of the behavioural proxy items did show a significant relationship with subjective irritability scores over time, and notably these included items related to argumentativeness (items 74 and 76a) and items related to outbursts (items 24 and 67). It is interesting to note that only one of these items (item 24) showed a significant interaction with age on irritability scores. Here, older participants (aged 21–36 years) showed a stronger relationship between item 24 (temper outbursts) and subjective irritability than participants aged 15–20 years. In particular, this relationship was strongest in those aged 21–25 years. In addition, item 24 did not correlate with MADRS scores in those aged 15–20 years, but did correlate with MADRS scores in participants aged 21–36 years, and this correlation was strongest in those aged 21–25 years (see Fig. 1).

This is particularly interesting and noteworthy because item 24 relates to temper outbursts, which have been shown to decrease with age, especially through development years.^22,23^ In addition, it is typically assumed that behavioural outbursts are underreported by adolescents themselves, with most studies relying on parent and clinician-rated assessments of behaviour.^24,25^ The present findings suggest there may be developmental differences in the threshold required for temper outbursts to occur. In younger individuals, a potentially wider variety of triggers, beyond subjective irritability, lead to temper outbursts, whereas in older individuals, the potential range of triggers that may lead to temper outbursts, including subjective irritability, are more limited. Thus, in older individuals, temper outbursts appear to be more closely linked to subjective irritability because of a decrease in potential triggers. And, in younger individuals, temper outbursts appear to be a less reliable indicator of subjective irritability when compared with older individuals. This novel finding is particularly relevant clinically, because behavioural consequences are often relied upon when assessing irritability in adolescents. Therefore, the assessment of subjective irritability should also occur as part of an overall assessment of functioning and morbidity in adolescents with a mood disorder. Currently, no comprehensive and validated measure of subjective irritability exists, and therefore future research should focus on developing an accurate and reliable measure of subjective irritability to gain a comprehensive insight into this important construct so that its manifestation in mood disorders can be understood.

The findings of the present study are important, as they indicate that temper outbursts may be more closely associated with depressive symptoms in adults than in adolescents and young adults, and that reporting of both subjective irritability and temper outbursts in adolescents and young adults may capture a more comprehensive clinical picture of these concepts than that attained through parent or clinician-rated measures. Furthermore, other behavioural measures, which could be considered more extreme or severe, such as item 67 (having the urge to break or smash things), did not interact with age. Thus, the current findings suggest that temper outbursts are less closely related to subjective irritability in young people than older adults, but that the urge to carry out more severe acts of frustration do not differ in their relationship with subjective irritability according to age.

It is important to note that the SCL90 is not a direct assessment of the frequency or severity of each of the items listed, but rather how much the individual was bothered by the particular item in the past week. Thus, the relationship between the argumentativeness and temper outburst items to subjective irritability may not necessarily be directly related in terms of frequency and severity, but rather in the perception and interpretation of these events occurring. In other words, an individual that is bothered by being subjectively irritable, is bothered by the behavioural consequences of this irritability resulting from an awareness that these outcomes have occurred in a state of heightened frustration, or having a lowered threshold than normal for responding in a hostile manner.^1^ Furthermore, the behavioural proxy items regarding argumentativeness involve interpersonal conflict, which may be particularly relevant to the population examined – hence the use and potential benefit of IPSRT in mood disorders.^26^ It may be the case that the population examined in this study (those with mood disorders) are more likely to have negative perceptions of interpersonal conflict generally, and have less adaptive coping styles, either as contributors to, or as a result of, their psychiatric illness.^27,28^

These findings have several important implications for the future assessment of irritability. First, changes in subjective irritability may not be accounted for by changes in overall mood morbidity as measured by the YMRS and MADRS, although in older individuals, depressive symptoms appear to be more closely coupled with subjective irritability. Second, when assessing irritability directly, items assessing argumentativeness and temper outbursts may not be effective proxies in younger individuals; however, in adults aged ≥21 years, these proxies appear to be more closely coupled with subjective irritability. In individuals with mood disorders, it may be that items regarding interpersonal conflict, such as argumentativeness and temper outbursts, are related to subjective irritability and are a particular source of distress. Thus, these items may be utilised to comprehensively assess the impact of subjective irritability on functioning. Therefore, further research is needed to examine the interaction between the subjective experience of irritability and mood (i.e. depression or mania), and to determine whether this relationship changes – and if so, how – according to age.

### Limitations

This study has several limitations. First, the population sampled did not include a healthy control group to assess whether levels of irritability present in the sample differed from those of individuals not experiencing mood disorder symptoms. However, the mean scores of SCL90 at baseline and throughout the study were above 0, which suggests that irritability was at least a concern for the patients, and as this group has significant mood symptom severity at the beginning of the study, it is likely that these scores of irritability differed meaningfully from healthy controls.^20^ The fact that all patients received psychological therapy throughout the study may confound findings regarding the changes of scores of irritability and mood over time; however, the relationships between these measures is the focus of this study, and this was still able to be interrogated. Future studies should employ a healthy control group to assess whether there are qualitative differences between how irritability is experienced in the context of a mood disorder, and whether there is an interaction between subjective irritability and mood.

Second, the relatively small sample size in this study did not allow for more developmentally sensitive age groupings in which to examine the relationship between subjective and behavioural measures of irritability. Although the current study did find important relationships between irritability, mood symptoms and age, future studies with larger sample sizes across the same age range could examine how these relationships change from mid to late adolescence and into early and mid-adulthood. In addition, larger sample sizes would facilitate a closer examination of younger participants and the relationship between subjective and behavioural measures of irritability. As these constructs were found to variously linked across different ages, detailed examination with a larger representation of younger participants would further confirm this finding and the consistency of this relationship in young people.

Third, because this study focused on a young cohort to examine how the relationships between subjective irritability and mood symptoms change throughout neurobiological development, the study did not include older adults. Older adults, and in particular those aged >60 years, should be the focus of future studies examining irritability, as past studies have shown interesting relationships between irritability and the onset of cognitive decline and neurodegenerative diseases, such as Huntington's disease^32^ and dementia^33^. Therefore, as adolescence and young adults provided an opportunity to examine irritability through a period of significant neurobiological change, so too would examinations of the same construct in older adults.

Furthermore, although several measures of irritability were used in this study, it is possible that they did not comprehensively measure the construct and in the case of the self-report items, a clear definition of irritability had not been provided to participants. This is problematic, as evidence suggests that the general population often differ significantly in their definition of irritability,^5^ and therefore this item may have been interpreted in a number of ways. Therefore, future studies should include a standardised definition of the construct for participants to ensure reliable and consistent assessment of irritability.

In addition to the potential inaccuracies regarding the self-reporting of irritability, the other items within the SCL90 hostility subscale may also be inaccurately reported, particularly in the younger participants in the sample. This is because the SCL90 asks participants to report the frequency with which they have been bothered or distressed by the listed problems, not necessarily the frequency of the problem occurring at any point. This is problematic, as insight may be impaired in participants with mood disorders, and adolescents in particular may have compromised insight with regard to interpersonal conflicts.^27^ Furthermore, half the participants were receiving IPSRT, which specifically focuses on interpersonal stressors as contributors to acute periods of illness.^29^ This may have resulted in participants being particularly attuned to interpersonal stressors, as measured by the SCL90, and may have inflated the relationship between these stressors, such as arguments and temper outbursts, and subjective irritability. Therefore, future studies may include the use of ecological momentary assessments to more accurately assess the frequency of behavioural proxies for irritability, and may corroborate this information with parental/caregiver reports of behaviour.^30,31^

In summary, this study identified several novel relationships between measures of subjective irritability, mood morbidity and behavioural proxies for irritability in a population of young patients with mood disorders. These relationships are of key significance for several reasons. First, changes in subjective irritability were not significantly related to scores of manic symptoms, but were differentially related to scores of depressive symptoms according to age. This suggests that the subjective experience of irritability differs according to the emotional context in which it occurs. Second, several behavioural proxies for irritability, such as argumentativeness and temper outbursts, were related to changes in subjective irritability over time, although temper outbursts were not particularly significant in younger participants. Taken together, these findings suggest that subjective irritability plays a key role in mood disorders especially from adolescence to adulthood, and that this relationship varies according to age. Specifically, subjective irritability is more closely linked to temper and behavioural outbursts in adults, whereas in adolescents, behavioural outbursts occur more independently, and may be a less useful indicator of the experience of irritability.

Thus, future research examining mood disorders should assess for subjective irritability in this population, and focus on attempts to both measure and examine this construct and how it is modified in the context of mood.

## Data Availability

The data that support the findings of this study are available from the corresponding author, E.B., upon reasonable request.
